# The Cause of Cancer
**Die Bestätigung der künstlichen Züchtung des Krebserregers,* by Dr. Robert Behla.


**Published:** 1910-07-16

**Authors:** 


### SPECIAL ARTICLES.

### THE CAUSE OF CANCER.*

PROFESSOR BEHLA is so well known for his researches on the distribution of cancer that any communication from him calls for careful consideration.

In the present essay he brings forward many points in support of his thesis that cancer is an infective disease. He advances evidence drawn from its well-known local variations in frequency, from its familiar incidence, and from cases of cancer *à deux*; and postulates the theory that an organism—probably a myxomycetes—is the causative agent.

He commences by criticising Bashford's assertion that there is no evidence of endemic or family cancer in man, animals, or trout. He says that the fact that Bashford and other observers have failed to meet with instances of what can be regarded as infection is no stronger proof that an infective agent is not at work than the fact that in an epidemic of meat poisoning one person does not acquire it from another, but from the common agent causing the infection.

The fact that people can find no evidence of infection does not prove that an infective agent is not at work. It is possible to put mice with sporidosis in the same cage as healthy mice, who will remain healthy. Is sporidosis therefore not due to an infective agent?

After advancing other arguments of similar nature, he goes on to speak of the nature of the organism he supposes to be responsible for cancer. He suggests that it is of a symbiotic nature, producing no toxins, and that the tissue reaction varies in man and different species of animals, just as we know it to vary in the case of such organisms as the bacillus tuberculosis.

Professor Behla advances many arguments in support of his views—some indubitable facts, others such unsupported statements as that "cancer infection is usually only met with when an open cancerous ulcer is in question." And there are very many of us who seriously doubt whether cancer infection ever occurs at all!

In the statistical portion of the paper are many interesting figures which relate to the apparent steady increase in the incidence of cancer in Germany since 1877. This is followed by the usual discussion as to whether the increase is actual or only apparent owing to improved diagnosis and more careful registration, and leaves one in just as much, or rather as little, doubt as the precisely similar arguments which have followed the publication of precisely similar figures in all other civilised countries.

For the benefit of those who are still in any doubt on the subject it may be interesting to mention the case of Iceland, a country where up to a certain period there was remarkably little cancer (recorded). A sudden increase then took place, and has been maintained ever since. This sudden increase was subsequently found to exactly correspond with the arrival of several doctors sent by the Danish Government, who had hitherto provided no medical service in the island.

Professor Behla speaks of striking geographical differences in Germany itself, and then refers to the influence of district upon the tissue or organ attacked. He mentions the well-known facts (*sic*) that cancer of the mouth is very common in the Argentine, cancer of the penis in Japan, and that cancer of the abdominal wall is very common in India. He apparently is not aware that this only applies to a very small province of India (Kashmir), and that the cancer is directly associated with burns produced by the habit of wearing a kàngri or hot pot in contact with the abdominal wall. Such vague and ill-founded generalisations militate seriously against the value of Dr. Behla's writings.

Later on he states that damp, low-lying districts show an abnormally high cancer incidence. This has been recognised in England, and the explanation has been advanced that such districts, owing to the proximity of navigable rivers, tend to be rather urban than rural in character, and that the resulting density of population, the large number of hospitals, and more careful diagnosis and registration fully account for the larger number of cases.

But it will be news to most people that in England those who spend much of their time in woods—*i.e.* gamekeepers, etc.—are commonly affected. We are familiar with no evidence on this point, and it is questionable whether with such a sparse and scattered population as that formed by woodmen and gamekeepers any exact information could be obtained.

The second part of the essay deals with the actual causative factor of cancer. Dr. Behla strongly holds the opinion that the whole group of cancerous diseases is caused by a parasite, that this parasite has the power of influencing all sorts of cells, and that therefore the type of growth produced depends primarily upon whether the epithelial, endothelial, or mesoblastic cells are chiefly affected. He believes that the parasite is one of the myxomycetes, and that he has successfully cultivated it *in vitro*. It will be immediately realised that these views controvert the theory that the cancer cell is itself the parasite.

One of the great difficulties met with in entertaining the view that a single organism or class of organisms is responsible for malignant growths is the extreme diversity of the latter. It is true that previous to the discovery of the bacillus of tuberculosis there would have been much difficulty in accepting lupus and pulmonary tuberculosis, for instance, as the results of the activity of an identical organism. But the difference between a carcinoma and a sarcoma is of a very much more fundamental nature than that between lupus and phthisis, although it is true that certain observers have re-

* *Die Bestätigung der künstlichen Züchtung des Krebserregers*, by Dr. Robert Behla.

corded the occurrence of the two cell types side by side, or even the slow conversion of one (carcinoma) into the other (sarcoma).

Another difficulty in accepting a universal organism is that occasioned by the congenital tumours. It might also be thought that the simple tumours would give rise to difficulty, but, after all, the words simple and malignant are merely comparative, useful for clinical purposes, but of no great scientific value. Many so-called malignant tumours are relatively harmless, while some " simple " tumours are often very lethal.

In the case of congenital tumours Professor Behla suggests that his organism has gained entry to the germ plasm itself—an interesting suggestion that sets one thinking of the extraordinary difference of behaviour in these congenital tumours. Some remain stationary and relatively harmless, others slowly grow; still others take on all the clinical characters of malignancy. All this Dr. Behla easily explains by supposing a varying intensity of the same organism.

The bacteriological aspects of Dr. Behla's work it is impossible to discuss in any detail here. He claims to have cultivated his organism, and further states that its life history is in the nature of a cycle, during whose various epochs the organism takes on different forms, at one time having the appearance of a coccus, at another a vacuolated structure.

In fact, he endeavours to fit into this cycle the organism described as a coccus by Doyen, and the intracellular bodies described by Plimmer. He does not appear to have been successful in causing the formation of malignant tumours in animals by the inoculation of his organism, but explains the difficulty quite legitimately by suggesting that only at certain stages of its life cycle is the organism inoculable, and then only when the cells of the host are in a suitable condition to receive it.

The essay would have been more convincing if it had been reduced by three-quarters, and all the arguments, asseverations, and denunciations of those who claim to have discovered the parasite of cancer—and there are many of them—had been omitted.

We shall look forward with great interest to further stages in the complicated life cycle of this vegetable parasite of Dr. Behla's, but are not so convinced, as he says he is, that the cancer problem, in so far as ætiology is concerned, is now solved!

